# Exploring multiple job holding practices of academics in public health training institutions from three sub-Saharan Africa countries: drivers, impact, and regulation

**DOI:** 10.1080/16549716.2018.1491119

**Published:** 2018-08-01

**Authors:** Woldekidan Kifle Amde, David Sanders, Baltazar Chilundo, Etienne Rugigana, Damen Haile Mariam, Uta Lehmann

**Affiliations:** a School of Public Health, University of Western Cape, Cape Town, South Africa; b Department of Community Health, Eduardo Mondlane University, Maputo, Mozambique; c School of Public Health, University of Rwanda, Kigali, Rwanda; d School of Public Health, Addis Ababa University, Addis Ababa, Ethiopia

**Keywords:** Capacity development, case study, context, dual practice, Ethiopia, higher education institutions, moonlighting, Mozambique, public health, Rwanda

## Abstract

**Background**: The paper examines external multiple job holding practices in public health training institutions based in prominent public universities in three sub-Saharan Africa countries (Rwanda, Ethiopia, Mozambique).

**Objective**: The study aims to contribute to broadening understanding about multiple job holding (nature and scale, drivers and reasons, impact, and efforts to regulate) in public health training schools in public universities.

**Methods**: A qualitative multiple case study approach was used. Data were collected through document reviews and in-depth interviews with 18 key informants. Data were then triangulated and analyzed thematically.

**Results**: External multiple job holding practices among faculty of the three public health training institutions were widely prevalent. Different factors at individual, institutional, and national levels were reported to underlie and mediate the practice. While it evidently contributes to increasing income of academics, which many described as enabling their continuing employment in the public sector, many pointed to the negative effects as well. Similarities were found regarding the nature and drivers of the practice across the institutions, but differences exist with respect to mechanisms for and extent of regulation. Regulatory mechanisms were often not clear or enforced, and academics are often left to self-regulate their engagement. Lack of regulation has been cited as allowing excessive engagement in multiple job holding practice among academics at the expense of their core institutional responsibility. This could further weaken institutional capacity and performance, and quality of training and support to students.

**Conclusion**: The research describes the complexity of external multiple job holding practice, which is characterized by a cluster of drivers, multiple processes and actors, and lack of consensus about its implication for individual and institutional capacity. In the absence of a strong accountability mechanism, the practice could perpetuate and aggravate the fledgling capacity of public health training institutions.

## Background

It is evident from the literature that multiple job holding is a pervasive phenomenon in the public sector and in higher education institutions (HEIs) in many countries [1–5]. The practice is known by different names, such as dual practice, dual job holding, moonlighting, multiple job holding, dual employment, multiple employment, dual working, double work, and plural employment [5].

In multiple job holding, staff augment basic salaries by engaging in a wide range of academic and nonacademic activities within and outside their home institutions [6,7]. How the practice is viewed and judged varies. Many [6–8] recognize and understand the underlying reasons. A World Bank report points out, ‘With wages so low, it is difficult to condemn such behavior’ [8]. Altbach recognizes the significance of multiple job holding beyond the additional income, and mentions its contribution in expanding the scope of experience and expertise of educators [6,7].

At the same time, others [8,9] have argued that external multiple job holding is detrimental to the academic quality in HEIs, suggesting that ‘Faculty moonlighting is … one of the more serious problems faced by higher education in developing countries’ [8]. It is argued that the practice further undermines the already weak capacity of health professional training [10].

It is widely recognized that HEIs in sub-Saharan Africa lack critical inputs to fulfill their mandates of developing the next generation of professionals [11]. This holds true for public health training as well [12]. The region has one of the lowest numbers of public health academics globally, with one study suggesting that the continent has a similar total number of public health academics as a major public health school in the North [13]. Amde et al. (2014) argue that underlying the lack of national leadership and capacity for health workforce development is weak capacity in public health training institutions [12].

Brain drain and predatory practices of Non Gorvernmental Organizations (NGOs) in the health sector have received attention and criticism, for example in the context of Global Health Initiatives [14]. However, it appears that the phenomenon of brain drain in general, and multiple job holding in particular, in public health training institutions has received relatively little national and international attention.

This study investigates the phenomenon of multiple job holding in three HEIs in the context of debates about capacity for public health education in Africa. It explores drivers and reasons, impact, and efforts at regulating practices. It seeks to illustrate the complex balancing act HEIs perform in having to attract or retain staff against competition for expertise from international NGOs, meeting their academic mandates, and working within the constraints of public service regulations. The study aims to contribute to broadening our understanding generally about what perpetuates weak capacity at Public Health training institutions in Africa, and specifically about the practice of multiple job holding in public health training departments in prominent public universities in three sub-Saharan Africa countries, namely University of Rwanda (UR), University of Eduardo Mondlane (UEM, Mozambique), and Addis Ababa University (AAU, Ethiopia). The institutions represent the oldest and most prominent training centers in the respective countries.

This research is part of a larger project assessing mechanisms that influence the design and implementation of an African capacity development partnership of universities to strengthen national leadership and training capacity in health workforce development in sub-Saharan Africa [12].

## Methods

This paper uses a qualitative multiple case study approach, which facilitates understanding of multiple instances of a phenomenon across varying contexts [15–19]. The research seeks to examine external multiple job holding in public health training institutions across three countries towards a broader understanding of the phenomenon in sub-Saharan Africa.

Eighteen participants were selected purposively from the three institutions (UR, UEM, AAU) based on their affiliation to the African capacity development partnership for health workforce development. The informants were involved in teaching or management and included mainly academic staff [17], with a few holding senior management roles in the institutions, and one administrative staff [1]. Semi-structured interviews were conducted with key informants, exploring a range of matters related to the partnership for health workforce development in general, and external multiple job holding in particular. The first author conducted face-to-face interviews with key informants (eight in Ethiopia, five in Rwanda, and five in Mozambique) between June 2014 and March 2015.

Documents pertaining to multiple job holding were also reviewed, including institutional reports, senate legislation, institutional strategic documents, consultancy guidelines, and news reports. The review served to generate data that respond to the research question, specifically about efforts to regulate the practice.

The data gathered from multiple sources using multiple methods was analyzed thematically, with themes generated deductively from the research question and inductively from data [19–21]. Broad analytical themes that pertain to various aspects of the phenomenon were drawn from the research question: What are the practices and perception about external multiple job holding and regulatory mechanisms? Categories that relate to the aforementioned themes were induced from the data. Three of the authors (ER, BC, and DHM) are members of the three public health training institutions, and hence acknowledge drawing on and incorporating their experiences.

Ethical clearance to undertake the research was obtained from the Senate Research Committee of the University of Western Cape, South Africa. The researchers strove to ensure confidentiality and anonymity of participants by removing any identifying information, and using systematic codes to refer to respondents [22,23].

## Results

### Scale and nature of practice

External multiple job holding was a prevalent practice among the public health training institutions in all three study countries. The practice was common among both senior and junior staff. Academic staff in the public health training institutions sought additional academic or nonacademic employment outside the home institution in the form of consultancies, clinical practice, provision of on-the-job training, teaching in other HEIs, commissioned research, etc., which happened with or without the knowledge/approval of the training institution.

One of the most commonly reported external multiple job holding opportunities was accepting teaching positions in the booming private HEIs. While this was widely practiced and popular in all three countries, according to one of the reviewed resources [24], Mozambique recently introduced a ban against academics in public HEIs teaching in other HEIs – a ban that met with substantial criticism: ‘… many higher education institutions and lecturers are unhappy [with the ban]…. Critics said the movement of teachers was helping to address the problem of shortages of quality academic staff and was augmenting low academic salaries’ [24].

One key informant suggested that the ban was not only criticized but also rarely observed, and the practice persisted. In Ethiopia, faculty also taught in the new public universities, which did not have adequate teaching staff of their own.

With respect to acting as consultants, key informants in Ethiopia and Mozambique suggested that staff were at liberty to seek and engage in consultancy without the knowledge/approval of the institution.

Clinical practice was also cited as one of the external engagements among academics. The institutional reports showed that the majority of academic staff had clinical backgrounds (66 and 97 percent in the Ethiopian and Mozambican cases, respectively).

### Drivers and reasons

Different factors at individual, institutional, and national levels, such as low salary, poor working conditions, and poor incentive structures, were reported to underlie and mediate the practice. These factors are discussed below.

#### Poor working conditions

Many key informants from the public health training institutions characterized their experience as educators in their respective public institutions as increasingly demanding and alienating. The institutions have relatively small staff complements considering the growing number of undergraduate and postgraduate students. As depicted in Table 1, in 2017 the permanent staff numbers in the Ethiopian and Rwandan cases seem comparable (42 and 48, respectively). The institution in Mozambique had only 14 permanent staff, and was found to rely more on contract staff than the other institutions (eight in Mozambique, and two each in Ethiopia and Rwanda). The number of students enrolled in both undergraduate and postgraduate programs in Ethiopia was the highest of the three institutions, perhaps due to the ‘flooding strategy’ the country has been pursuing recently.

**Table 1.** Profile of public health training institutions.

Institutional profiles		UEM	UR	AAU
Year department established		1962	2001	1964
Number of academic staff in 2017	Permanent staff	14	48	41
	Contract staff	8	2	2
Gross salary per month for different staff levels in 2017^1^	Full Professor	1078 USD (32,360,00 MZN)	2240 USD (1.870.731 RWF)	880 USD (20,245 ETB)
	Assistant Professor	674 USD (20,240,00 MZN)	1847 USD (1.542.447 RWF)	583 USD (13,420 ETB)
	Lecturer		1115 USD (931.500 RWF)	455 USD (10,470 ETB)
Number of students in 2017	Undergraduate	268	222	4000^2^
	Master’s	88	83	283
	PhD	0	12	45

^1^Does not include allowances, Exchange rate: 1USD = 835 RWF/ 23 ETB / 30 Meticais.^2^No undergraduate public health program but courses taught to medical and other health sciences students.

It was evident that many experienced ever-growing workloads, as a result of a proliferation of programs and increasing enrollment targets (See Table 1).

*… When we try to calculate workload according to the Excel sheet they [the university] provide us, you see that for a lecturer for example, you must not go beyond 900 hours. When I try to fill this I find 1800 … or 2500 [hours]. You find that it is a bit crazy. That is evidence that we are not* enough. (Senior academic staff member, Rwanda)

*This is the oldest school in public health. … But today from 21 medical departments, you will only find eight or nine professors. They may not have four assistant professors. But you look at the student number; it is massive at all levels including PhD. If we don’t find solutions [in a timely manner], it breeds further and complicated problems.* (Senior academic staff member, Ethiopia)

#### Poor incentive structure

Key informants reported that the demands imposed on educators were not commensurate with the salaries they earned, which compared unfavorably with salaries paid in NGOs, state-owned enterprises, or the private sector. They asserted that their salaries covered only a portion of their living expenses. A key informant from Rwanda described the situation as being grossly unsatisfactory:

*In our country [the] academic sector is not attractive. … I could [earn] two times, three times my salary if I [went] to [the] Ministry of Health or [an] NGO.* (Senior academic staff member, Rwanda)

Grievances concerning their salaries on the part of educators were described as longstanding and substantially unchanged, despite many pledges by their respective governments. *A*cademics expressed their pessimism about the willingness and ability of government to introduce significant increases in their salaries.

*I don’t think the state/government is ready to pay for staff to be full time here at the university. I don’t think they are ready for that. They [would] have to make it [salary] four [times] what it is now*. (Senior academic staff member, Mozambique)

*Government claims it is going to fix this, and has taken ownership of the problem. We don’t see anything*. (Senior academic staff member, Ethiopia)

*The problem is massive. It is also related to [the country’s] development. Everything has been tried to reform the civil service system*. (Senior academic staff member, Ethiopia)

A closer look reveals that academics earn comparable base salaries to their peers in pubic sector/civil services, but some participants emphasized that benefits in the public sector are bigger than in HEIs. The grievance, however, seems to be triggered in comparison to salaries in state-owned enterprises, or NGOs or international agencies, to which HEI staff members’ salaries are inferior.

*University remuneration used to be comparable to all the prestigious profession[s] such as airlines and telecommunications. This declined over time. University lecturers have to do consultancy.* (Senior academic staff member, Ethiopia)

*For the same level of [degree [master’s]) the salary of [the] public sector is almost the same, but for the public sector, they have additional advantages: transport, communication … so [in] the end, the public sector officer earns almost twice the academic.* (Senior academic staff member, Rwanda)

With the current salary levels and working conditions, attracting qualified staff was reported to be daunting. Many institutions struggle to fill vacancies in the context of high turnover rates. It was evident from the responses that faculty members often left their posts to join international agencies at home or abroad:

*It [the salary] is not something [that attracts] people. We have lost different qualified staff in [recent] years. Many colleagues went to Europe… some are in Brazzaville, Geneva, US. This is why we are suffering from [a] shortage of qualified staff’* (Senior academic staff member, Rwanda)

Most of the turnover was characterized as causing dysfunction, with the most senior and most qualified, who are hard to replace, being the most highly sought after, especially by international agencies/external development or training partners. Unable to attract senior staff, institutions thus resort to hiring junior staff to replace the departing seniors:

*As if it is a resignation letter, the moment they [academics] get their professorship they leave the institute for a local or international post. They can get a better salary nationally. [If] they join [international agencies] … there [are] lots of them… Most of them are staffed with former university staff. … We have [now mostly] fresh graduates, who could not teach [as educators]. We all started that way. The difference is that we had mentors…. In most universities [currently], we don’t have that [mentorship]*. (Senior academic staff member, Ethiopia)

Key informants from universities suggested that staff found it hard to turn down opportunities that come their way due to associated financial benefits and the unpredictable nature of opportunities for multiple job holding. As a result, they were inclined to take up as many external jobs as possible:

*I have to run after money, unfortunately, to survive. My salary is definitely not enough. It does not pay half of my bills. I have to find [additional income]. … Generally it [my salary] pays for my house rent, and maybe one of my kids’ school [fees]. No more than that.* (Senior academic staff member, Mozambique)

### Impact

#### Benefits to academics

Key informants emphasized that training institutions were able to attract and retain staff not for the salary, but because of the opportunities they afford for generating additional income, through involvement in training, research, and outreach programs. Although a few mentioned intrinsic benefits like recognition and reputation as major attractions, the possibility of being able to engage in multiple job holding seemed to be regarded as inherent to, and the principal advantage of, working in the university, compared to working in the private sector or an NGO:

*I like the [university] employment because it is flexible. I can do something else. Even without any authorization here.* (Senior academic staff member, Mozambique)

*One of the advantages of working [in public higher education] is that you have the freedom. … As long as I keep [the] class schedule, and secure my responsibility, no one is bothered where I am. … That is the biggest incentive. The freedom. If that was not there, no one [would want] to stay.* (Senior academic staff member, Ethiopia)

#### Risks to academic institutions

Despite the above claims that multiple job holding was beneficial and had little negative impact on the core responsibilities of faculty and institution, some key informants argued otherwise. They suggested that the discretion faculty have, and the marketability of their expertise (in the booming NGO sector, private HEIs, and private hospitals) is a major distraction from their teaching and research responsibilities.

*The low payment spur[s] staff to chase consultancy service[s], which is detrimental to their teaching practice … Some staff [are] said to only show up a few times [in] the whole year, and [try] to fit a semester course [into] three days*. (Senior academic staff member, Ethiopia)

According to participants in this study, research and publications particularly suffer due to external multiple job holding, and there is little incentive for academic staff to publish. Some staff even dedicated their sabbaticals to taking up short-term employment in the private or NGO sectors.

This seems to be further aggravated by poor motivation, which was reportedly related partly to the lack of support faculty receive for engagement in research and publication in the training institutions. The universities did not appear to have any specific time-bound expectations regarding research publications, and no associated incentives either. When asked if the heavy involvement of academics in consultancy work interfered with responsibilities such as teaching and publication, one key informant replied:

*As teaching is well planned [it doesn’t affect it so much], [but] it is undermining publishing … We have a lot of data, sometime[s] some people don’t have time to go through [the data and publish].* (Senior academic staff member, Rwanda)

### Efforts to regulate

#### Broad policy

Each institution has a formal policy/legislation/strategic document (see Table 2) that makes provisions for academics to engage in external multiple job holding on condition that it does not interfere with the primary job responsibility [25–27]. A closer look at the policies shows that they all emphasize the importance of realizing the institutional mandate and protecting the reputation of the institution. However, except in the case of the Rwandan institution, where academics are required to seek permission from their home institution prior to involvement in external jobs, the policies were found to be too broad and vague to operationalize or serve as an accountability mechanism. Key informants across all contexts reported that regulatory mechanisms were either not clear or not enforced. Even in the case of the Rwandan institution, there were reports of undeclared participation in external multiple job holding.

**Table 2.** Institutional policy about external multiple job holding.

UEM, Mozambique	UR, Rwanda	AAU, Ethiopia
*‘It is the responsibility of departments and research centres to promote the transfer of knowledge, produced by research, to decision-making bodies, the productive sector and society, in general, through extension activities. For this, it is urgent … [to] regulate extension, consulting and service activities by the units and centres, as well as by teachers and researchers, providing incentives that stimulate the teacher and the researcher’*. [25]	UR recognizes the benefit of its staff undertaking consultancy services … to enhance financial capability of the university and staff as well as contribute to the development of the core activities of the university particularly research. ... UR staffs are encouraged to engage in consultancy as a valued and legitmate activity. … University employees are permitted to undertake … consultancy activity … with approval of the College Principal. [26]	*‘No academic staff shall undertake any outside activity, which may impair his usefulness to the University or conflict with his duties. The provisions of this shall, however, not be deemed to constitute a bar on an academic staff from participating in social organizations, civil societies, professional associations or consultancy services.”* [27]

#### Weak practice

Key informants reiterated that freedom and flexibility to undertake external work came with certain responsibilities, and staff were expected to deliver on their basic responsibilities to students, the university, and donors. However, there were challenges in this regard, and the extent to which faculty lived up to this expectation varied, depending on self-regulation:

*There is no limit to how much work you can do … as long as your routine work is not affected. … The practice of the staff in this regard depends on personal commitment. There are those who do it well, and those who don’t.* (Senior academic staff member, Ethiopia)

There was little effort in terms of supervision and enforcing regulations. The tacit tolerance may have emanated from appreciation of the poor incentives and working conditions under which academics operate, or the substantial funding the institutions generate in terms of research or contract overheads.

*In my department they [junior staff members] are quite free. I am not telling them “you are free to do this.” I know they are finding ways. I am not controlling their entire time presence; I just want results for my department*. (Senior academic staff member, Mozambique)

The institutional arrangements to generate funding through high overheads, which are often not matched with adequate administrative support, have led some academics in Rwanda and Ethiopia to undertake consultancies without the knowledge of the home institution. This is done with the intent to pay relatively low overheads, or circumvent the perceived lack of administrative support from their home institution, such as expediting release of project funds.

*[Since 2015] an academic staff [member] involved in a consultancy should [receive] only about 32 percent of the amount [paid for the] consultancy: the remaining [goes to] taxes and [the] university … meaning that motivation [for] doing consultancy is decreasing unless some faculty apply to consultancy [posts without declaring this] at [the] university level.* (Senior academic staff member, Rwanda)

## Discussion

This study contributes to the body of literature on multiple job holding practice by highlighting the complex nature of the phenomenon involving multiple processes and actors with differing interests. External multiple job holding practice among academics in public health training institutions has multiple drivers, evokes contrasting views about its impact on academics’ or institutions’ capacity and performance, and raises the issue of the viability and implications of strategies to regulate the practice.

This study has shown that external multiple job holding is a longstanding and ubiquitous practice among academics across all three public health training institutions considered. Findings from the study illuminate different aspects of the practice. Locating academics in the institutional and local realities in which they operate was found to help advance understanding of the nature and pervasiveness of the phenomenon in the respective settings. The institutions under examination are experiencing growing enrollment, poor incentive structures, and poor retention and attraction of qualified staff (see Table 1).

Perceptions regarding academics’ engagement in external multiple job holding practice (teaching, clinical practice, and consultancy) varies. The excessive and unchecked external engagement by some academics is considered by most to undermine the capacity of home institutions to accomplish their core responsibilities. Conversely, positive sentiments towards the practice exist in relation to the additional income it affords academics, which some even credited with attracting or retaining employment of qualified academics in public universities. Furthermore, some highlight the contribution of the practice towards promoting the profiles of academics and institutions.

One of the important findings of the research is that it reveals that few accountability mechanisms exist or succeed with respect to the practice. The broad guidelines the institutions have in this regard (see Table 2) are not observed or enforced effectively, leaving academics to self-regulate. This high level of tolerance academics enjoy in the institutions seems to be strongly related to the following conditions: appreciation of the poor working conditions and payment in the institutions; inability of the institutions to enforce restrictions under these circumstances without losing academics to better-paying international or local NGOs; or reliance on the substantial funding the institutions generate in terms of research or contract overhead.

Academics seeking multiple job holding to supplement their meager salaries is a scenario that is well documented in the literature [28,29]. The positive views and benefits of external multiple job holding, similar to the ones mentioned in this study, are also highlighted in the literature. Multiple job holding helps increase income and expand the scope of experience and expertise of educators, which contributes to enhancing the quality of academic engagement in the main institution [7].

By contrast, the study reveals that if left unchecked, academics may abuse the privilege of participating in external multiple job holding, which undermines faculty performance and the realization of institutional mandates. The practice was reported to particularly affect academics’ availability for and performance in teaching responsibilities, and to reduce the drive for research productivity. This supports observations made in other studies about the negative implications of external multiple job holding, with many characterizing any additional job holding outside the institution as detrimental to the academic quality in the public institution [6,7,28–30]. ‘[Moonlighting] affects public universities in an undesirable way [… resulting in] low or non-existent publication records, and little time to supervise students and prepare quality lecturers’ [28].

Literature also supports our findings about the excessive engagement of academics in multiple job holding practice in response to the uncertainty/scarcity of external multiple job holding opportunities and the pressure of maintaining the standard of living made possible by additional income from multiple job holding. A multi-country study on multiple job holding reported: ‘For many academic staff in most of the countries … a middle-class income requires additional employment’ [7] in a context wherein HEIs were considered part of the public sector, which meant working under poor conditions and low salaries [7,9].

The study revealed that the institutional arrangements in the public universities afford faculty members the freedom and time to engage in external multiple job holding. Similar claims were made in two studies conducted in Ghana [31] and Cameroon [32]. ‘Apart from the financial motive that drives an individual’s decision to moonlight, the engagement of moonlighting on account of lower working hours in the individual’s main job could be a symptom of visible or time-related underemployment’ [31].

As reiterated in this study, the culture of tolerance towards the practice was what drives many to seek employment or continue to work for the institution. Against this background, putting in place appropriate strategies to regulate the practice is a complex undertaking. A group of experts from 13 countries, who deliberated on ‘perils and promises’ facing HEIs in developing countries, identified as major challenges the practice of unregulated external multiple job holding and lack of accountability mechanism thereof [8].

Contestations around ways to address the phenomenon or its negative implications are common in the literature. Altbach et al. (2012) call for recognition of the multifaceted and complex nature of the challenges facing the academic profession, and the need to address issues like external multiple job holding in a concerted manner [7]. In line with this, some measures to address the low pay in public institutions were also criticized for being too limited, using the wrong indicators, or focusing more on research than teaching, perhaps due to the difficulty of measuring the latter [24,29].

Literature on external multiple job holding among health workers also warns against restrictions such as banning outside professional practice in the public health sector, in the context of low salaries and staff possessing highly marketable skills [33]. ‘Preventing professionals from undertaking private practice may induce them either to leave the sector entirely or migrate overseas’ [33].

The state of brain drain and multiple job holding in public health training institutions evidenced in this study reflects the situation of clinicians in many respects [3]. Firstly, a significant proportion of academics have clinical backgrounds, with high demand for their expertise in academic or nonacademic work, including clinical practice. Secondly, the private sector and NGOs, both national and international, play a prominent role in enticing senior faculty, and contributing to the high turnover rate and vacant positions. This has been substantiated by previous studies, such as one in Ethiopia that highlighted an overall turnover rate of 92.8 percent within a 20-year period in the medical faculty, with the public health department being one of the most affected [34].

In the context of weak health systems and very inadequate public health capacity in the countries regarding trained personnel, it is ironic that external agencies in Low and Middle Income Countries (LMICs), which are primarily meant to engage in health system strengthening, inadvertently undermine the national ability to strengthen public health through training and research.

The findings of this research reflect the diversity of perceptions and experience of participants about various facets of external multiple job holding practice in the three public health training institutions. However, the findings cannot be generalized to other public health institutions in the countries. Future research should focus on measuring the magnitude of practice and its multifaceted reasons and implications, and assess the effectiveness of existing approaches targeting the practice to inform future intervention.

## Conclusion

External multiple job holding practice is a complex phenomenon characterized by multiple drivers, processes (formal or informal), actors (internal or external to the main institution), and divergent interests. The practice is also marked by a lack of consensus about its implications for individual and institutional capacity, and the nature and scope of regulatory mechanisms targeting the practice. In the context of a complex set of interacting drivers, public health training institutions make provisions for academics to be involved in the practice as a strategy to attract/retain staff or generate funds/reputation for the institutions. In the absence of a strong accountability mechanism, the practice can perpetuate and aggravate the fledgling capacity of public health training institutions.

## Paper context

Public health training institutions in sub-Saharan Africa assume a prominent role in training the next generation of health professionals and addressing the myriad health challenges facing countries. These institutions, however, face multifaceted challenges in fulfilling their mandates, and efforts to strengthen capacity are mediated by a range of factors. This study seeks to gain an understanding of the capacity implications of external multiple job holding, which is a widespread phenomenon for public health training institutions in public universities in the region.
